# The First Clinical Use of Augmented Reality to Treat Salivary Stones

**DOI:** 10.1155/2020/5960421

**Published:** 2020-07-09

**Authors:** Anna Lysenko, Alexandra Razumova, Andrey Yaremenko, Rustam Mirzakhmedov, Anna Zubareva, Marina Chibisova

**Affiliations:** ^1^Department of Maxillofacial Surgery, Research Institute of Dentistry and Maxillofacial Surgery, Russia; ^2^Department of Dental Surgery and Maxillofacial Surgery, First Pavlov State Medical University, Russia; ^3^Create Unique 3D Filters, Russia; ^4^Department First Pavlov State Medical University, Russia; ^5^Department of Radiology in Dentistry, Private Educational Institution SPb INSTOM, Private Educational Institution St. Petersburg Institute of Post-Graduate Dentistry, Russia

## Abstract

In this study, we report our first experience of applying the concretion visualization method using augmented reality technology. A clinical case of a new surgical intervention on the parotid salivary gland with the localization of salivary stone in its parenchyma is considered. During additional diagnostics, it was found that the size of the concretion exceeds 5 mm which did not allow us to use the endoscopic technologies. That was the reason for the choice of surgical intervention external access using salivary stone visualization with the help of augmented reality. The preoperative procedures included making the upper jaw cast model, fitting the model and individual mouthguard with an X-ray contrast marker and marker slot. In addition to this, computed tomography of the head and neck using a mouthguard was made. During surgery under general anesthesia with nasal intubation, the mouthguard together with the marker is installed in the patient's mouth and the surgeon puts on the glasses to visualize the stone image in place of its localization. This method enables to visualize the salivary stone on all surgery stages no matter what type of approach is used or performing hydropreparation. That is why using the augmented reality appears promising and is to be studied further.

## 1. Introduction

Salivary stone condition is a polyetiological pathology of the salivary glands. The primary factor of stone formation is still not discovered. That is why the main method to treat salivary stones is to remove the salivary stones or the gland extirpation [1].

Most often a stone is localized in the submandibular salivary glands, rarer in the parotid salivary glands with the incidence reported up to 20% [2]. Surgical intervention in this area can lead to both operative and postoperative complications such as the facial nerve branch damage or formation of fistulous tract. That is why development of new organ preserving methods of salivary stone treatment is an important issue of the contemporary maxillofacial surgery. Using modern methods such as sialoendoscopy is not always available due to the very expensive equipment needed and a specialized training to be performed [3]. Today, the augmented reality technique is steadily implemented in our everyday life and is integrated with the picture of reality not distorting it [4]. In February 2018 in the Almazov National Medical Research Center, a memorandum about establishing a national project called “Digital Healthcare” was signed. It is aimed at implementing and developing digital technologies in the Russian Federation healthcare [5]. That is why it enables us to implement its potential in medicine as well, particularly, in the maxillofacial surgery. The ability to combine the reality and virtual reality with the first one prevailing as well as the total interactivity of all “artificial” objects and their 3D presentation, the ability to evaluate it from all aspects, and their interaction with the real world allow us to work out the newest surgical method in case of stone removal from the parotid salivary gland parenchyma.

## 2. Materials and Methods

A female patient М. 33 years old presented on the Oncology Department #8 (Maxillofacial Surgery Department), I.P. Pavlov SPbSMU, with pain and swelling in the preauricular area from the left side, becoming enlarged while eating.

### 2.1. Past Medical History

The first time the patient noted the pain syndrome and the parotid gland enlargement from the left side was 10 years ago during the meals. During this time, the symptoms resolved spontaneously. A year before the admission to the Oncology Department #8 (Maxillofacial Surgery Department), I.P. Pavlov SPbSMU, the patient had undergone in some other hospital an in-patient ultrasound examination of the salivary glands showing the stone. They performed only intraoral drainage in the Stenonov duct projection with its dissection. Though, after the inflammatory process subsided, the pain and enlargement of the parotid salivary gland did not resolve. The patient presented to the Oncology Department #8 (Maxillofacial Surgery Department), I.P. Pavlov SPbSMU, for surgery arrangement.

### 2.2. Status Localis

Examination showed hyperplasia of the parotid salivary gland from the left side. The skin of the preauricular masticatory area from the left side is not changed in color. The parotid salivary gland is dense but painless on palpation. While massaging the parotid salivary gland, there is a little amount of pure saliva secreted. The oral cavity is sanitized. The oral mucous membrane is pink, smooth, and shiny. The Stenonov duct opening is slightly deformed; there is a scar changed mucous membrane of the cheek.

After the basic examinations, additional diagnostics were prescribed.

**Figure 1:**
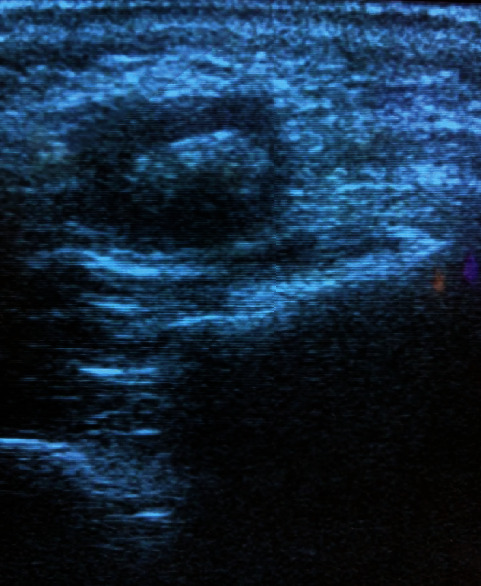
Echography of the patient M. left parotid gland showing the hyperechogenic mass with 3 × 5 mm acoustic shadow.

The echography picture during the large salivary gland ultrasound examination showed dilatation of the outflow duct; there, a hyperechogenic mass with 3 × 5 mm acoustic shadow was visualized (Figure 1).

There were no signs of purulent liquefaction of the parotid salivary gland found. Considering the stone size and scar deformation of the duct as well as its localization, we decided to refuse from removing the stone using a wire basket and sialoendoscope and to apply the method of augmented reality to visualize the salivary stone.

**Figure 2:**
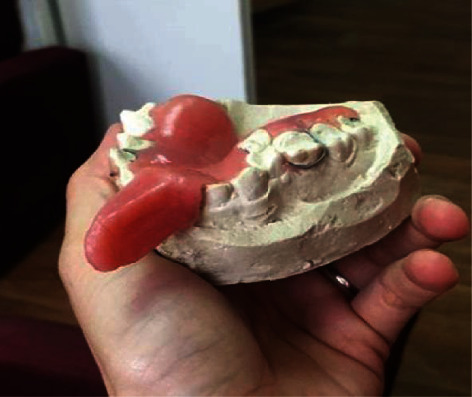
Individual occlusal splint with a radiopaque marker.

Preoperatively, the cast model was made, and the individual mouthguard was produced having an X-ray contrast marker with the marker slot (Figure 2).

A spiral computed tomography with the individual mouthguard was performed to transmit the image to the augmentation reality glasses.

**Figure 3:**
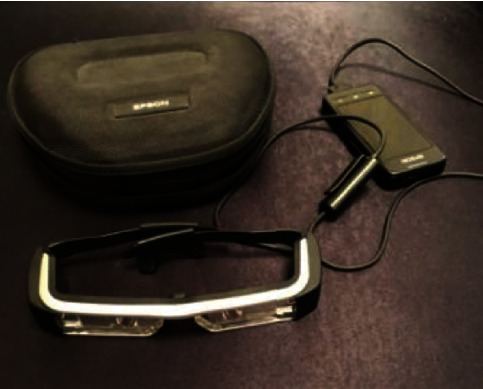
Augmented reality glasses (company: Epson Moverio BT-200, eyepieces resolution 960 × 540 px, weight: 88 g).

The surgery is performed under endotracheal anesthesia with nasal intubation. The patient had her individual mouthguard on the lower jaw. After that, the surgeon put on the AR glasses (Figure 3).

**Figure 4:**
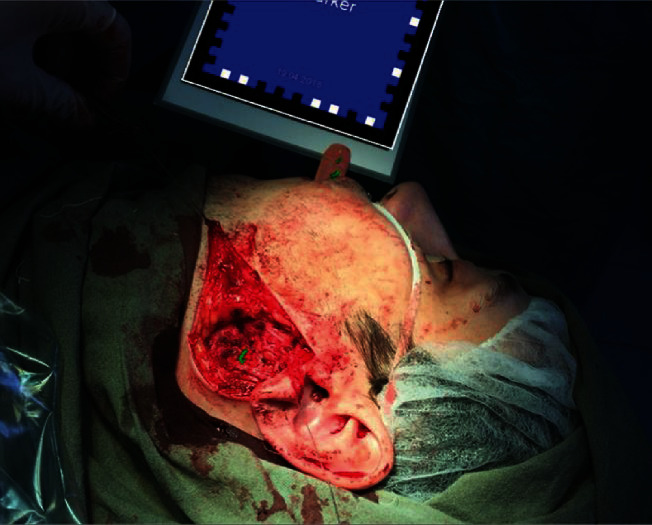
Salivary stone removal surgery using augmented reality techniques (the concretion is located in the surface layer of the parotid gland parenchyma).

In the real-time regimen, the surgeon was able to see the virtual picture of the stone localization in the parotid salivary gland (Figure 4).

After that, an S-shaped incision [6] in the preauricular region of the skin and subcutaneous hypoderm was made; the flap was made and the capsule incised. The part of the duct where the stone was visualized was bluntly selected and incised and the stone was removed. Then, a stent was installed and fixed in the oral cavity to prevent stricture formation of the duct. The wound was sutured layer by layer.

## 3. Discussion

In this clinical case, the technique of using augmented reality to visualize the concrement during the surgical intervention of salivary stone removal using external access is shown. At present, minimally invasive methods for removing parotid gland salivary stones exist and these methods are performed using sialoendoscopy with the wire basket or laser, chemical and shock wave lithotripsy. However, if there is a narrowing, stricture, or cicatricial deformity of the salivary gland duct, using of these methods is not possible. The technique we are developing allows to change the view on surgical treatment of patients with salivary stone disease. It is based on thorough preoperative preparation and an individual approach to each patient so an individual mouthguard with a tap is made and computed tomography is also performed. Augmented reality technology allows to visualize the concrement in the operating field and shows an accurate localization of it in the salivary gland duct. Thus, augmented reality technology can reduce the time of surgery and force the postoperative recovery period. However, it is not possible to calculate the depth of the concretion and it is also necessary to reduce the localization errors of the salivary stone virtual image. If these disadvantages are eliminated, it is possible to reduce the length of the incision and the volume of surgery to the minimum possible.

## 4. Conclusion

The use of the augmented reality technique for concretion visualization during the surgical intervention of salivary stone removal by external access was effective, and there was no any postoperative complications. However, randomized controlled clinical trials with longer follow-up periods should be conducted in order to compare the effectiveness of this technique, its injury rate, and the possibility of using it in the daily practice of a maxillofacial surgeon.
